# The balance between traffic control and economic development in tourist cities under the context of COVID-19: A case study of Xi’an, China

**DOI:** 10.1371/journal.pone.0295950

**Published:** 2024-01-30

**Authors:** Wang Xiang, Zezhi Wang, Xin Pan, Xiaobing Liu, Xuedong Yan, Li Chen

**Affiliations:** 1 Hunan Key Laboratory of Smart Roadway and Cooperative Vehicle-Infrastructure Systems, Changsha University of Science and Technology, Changsha, Hunan, China; 2 State Grid Hunan Electric Power Company Limited Economic & Technical Research Institute, Changsha, Hunan, China; 3 Hunan Key Laboratory of Energy Internet Supply-demand and Operation, Changsha, Hunan, China; 4 School of System Science, Beijing Jiaotong University, Beijing, China; 5 Key Laboratory of Transport Industry of Big Data Application Technologies for Comprehensive Transport, Beijing Jiaotong University, Beijing, China; 6 School of Traffic and Transportation Engineering, Central South University, Changsha, Hunan, China; University of Naples Federico II: Universita degli Studi di Napoli Federico II, ITALY

## Abstract

Selecting an appropriate intensity of epidemic prevention and control measures is of vital significance to promoting the two-way dynamic coordination of epidemic prevention and control and economic development. In order to balance epidemic control and economic development and suggest scientific and reasonable traffic control measures, this paper proposes a SEIQR model considering population migration and the propagation characteristics of the exposed and the asymptomatic, based on the data of COVID-19 cases, Baidu Migration, and the tourist economy. Further, the factor traffic control intensity is included in the model. After determining the functional relationship between the control intensity and the number of tourists and the cumulative number of confirmed cases, the NSGA-II algorithm is employed to perform multi-objective optimization with consideration of the requirements for epidemic prevention and control and for economic development to get an appropriate traffic control intensity and suggest scientific traffic control measures. With Xi’an City as an example. The results show that the Pearson correlation coefficient between the predicted data of this improved model and the actual data is 0.996, the R-square in the regression analysis is 0.993, with a significance level of below 0.001, suggesting that the predicted data of the model are more accurate. With the continuous rise of traffic control intensity in different simulation scenarios, the cumulative number of cases decreases by a significant amplitude. While balancing the requirements for epidemic prevention and control and for tourist economy development, the model works out the control intensity to be 0.68, under which some traffic control measures are suggested. The model presented in this paper can be used to analyze the impacts of different traffic control intensities on epidemic transmission. The research results in this paper reveal the traffic control measures balancing the requirements for epidemic prevention and control and for economic development.

## 1. Introduction

The novel coronavirus in 2019 (COVID-19) has evolved as a global pandemic. As of October 31, 2022, the cumulative population of confirmed cases had exceeded 630 million, and the cumulative population of death-from-disease cases had exceeded 6.5 million, all throughout the globe [1]. While posing a menace to life safety, the outbreak of the global pandemic has brought about huge impacts on economic development. Economists estimated through analysis that the global economic growth rate would drop by 1.6% by 2020 and 3.2% by 2021 [2]. During the period of COVID-19, tourist cities were suffering a huge impact [3]. The term ‘tourist cities’ refers collectively to a class of cities that possess unique natural landscapes or humanistic resources and other unique resources, with potential appeal to and certain accommodation capacity for tourists, and that are centered on scenic areas and spots and based on the tourist industry, with a tourism output value exceeding the urban GDP by 7% [4]. Under the context of COVID-19, the traffic limitation both home and abroad has posed a significant challenge to the tourism industry, leaving a substantial impact on the evolution of tourist demands [3]. Under the impact of COVID-19, in 2020 the number of international tourists dropped by 74%, export proceeds decreased by 1.3 trillion dollars, and 100 million to 120 million tourism jobs were confronted with a risk [5]. Making a choice between epidemic control and economic development has become a harsh challenge for cities with well-developed tourism [6].

In order to block the transmission of COVID-19, most countries adopted various epidemic prevention and control measures [7]. Lessening mobility helps reduce virus propagation [8]. Traffic control policies would affect population mobility, thereby limiting the spread of COVID-19 [9]. At present, all national governments have adopted the corresponding traffic control measures to curb the epidemic development and achieved certain success in epidemic prevention [10]. The strict policies of physical distancing and the stay-at-home order have been considered effective methods [11]. Meanwhile, the pathogenicity of some COVID-19 strains has declined after mutation [12]. Despite the emphasis of all these previous studies on the effectiveness of traffic control measures under epidemic, unreasonable epidemic prevention and control measures could negatively impact the society and economy. Excessive epidemic prevention only seizes the contradiction between epidemic spread and epidemic prevention and control but neglects the contradiction between people’s real-world production and livelihood and the unreasonable anti-epidemic measures [13]. In view of the effectiveness of any control measure with respect to epidemic prevention and control, therefore, it appears extremely crucial to balance epidemic prevention and control and the goal of economic development for the two-way dynamic sustainable development of epidemic prevention and control and economic and social progress.

To explore the above issue, this paper proposes a SEIQR model considering population migration and the propagation characteristics of the exposed and the asymptomatic, including the traffic control intensity in model building. Based on the determination of the function relationships between control intensity and the epidemic target as well as the tourist economy target, the multi-objective optimization algorithm NSGA-II is employed to get a traffic control intensity that can balance epidemic prevention and control and economic development, via the thought of combinatorial optimization, and suggest the corresponding traffic control measures. The primary contribution of this study builds upon previous research and enhances the virus transmission model by incorporating real-world virus transmission dynamics while considering population mobility and the characteristics of asymptomatic carriers. By introducing a traffic control intensity coefficient into the infection rate, different traffic control intensity can be simulated, providing valuable insights for government pandemic control efforts and laying the groundwork for subsequent multi-objective optimization. Historically, research has primarily focused on the effectiveness of traffic control in pandemic containment. However, in addressing the challenge of balancing pandemic control and economic development, this study employs a multi-objective optimization approach to explore traffic control measures that achieve such equilibrium. This research mitigates conflicts between control measures and economic development, thereby avoiding the economic harm associated with excessive pandemic control. It facilitates the recovery and operation of society and daily life, effectively promoting a bidirectional dynamic synergy between pandemic control and economic development.

The rest of the paper is structured as follows. The next section provides a literature review. Section 3 presents the study area and data used, and details the research methodology. Section 4 presents the results and analysis. Section 5 provides the discussion. Section 6 provides the conclusion of this study.

## 2. Literature review

### 2.1 Research of epidemic transmission models

The characteristics of virus infection determine the structure of epidemic prediction models [14]. The virus propagation models researched by predecessors fall into two major classes. The first class are epidemic transmission models based on classical dynamics (e.g., the SIR model), dividing research objects. Through parameter fitting, the variations of each group of people throughout the epidemic development process can be described quantitatively [15]. This class of model has fully explored mathematical properties and scalability at the city level [16–18]. Based on the SIR model, scholars have optimized multiple types of model parameters to simulate the real transmission process of an epidemic by, for example, considering the latency period and time-varying spreading rate of the epidemic [19,20]. The quantitative SEIR model considering the infectiousness of the exposed based on the improved coefficient of infection rate can be better applied in traffic control strategies [21].

The second class are virus propagation models based on machine learning and other algorithms. These methods can predict epidemic transmission through learning and training based on the historical data [22]. Some scholars have built a combinatorial prediction model associating the dynamic model for epidemics with machine learning algorithms [23].

### 2.2 Research of traffic control and tourism economy under the context of COVID-19

Traffic control policies could impact population mobility, thereby limiting the spread of epidemic [9]. In view of the effectiveness of traffic control in epidemic prevention and control. Some scholars have proposed the traffic control policies with “risk level settings”, upgrading the risk level of regions with confirmed cases to medium or high [24] to block the epidemic transmission [25,26]. Some studies have statistically analyzed the tendencies of epidemic transmission before and after the implementation of three typical traffic control measures: road closure within the city, passenger transport outage, and community population mobility control [27]. With the improved model, one can simulate the epidemic development status of city agglomerations under traffic control measures such as first-level response to a significant public health emergency and traffic barring in epidemic-stricken areas [28]. The research on traffic control measures for epidemic prevention and control elucidates the regulatory efficacy of various traffic management interventions, thereby furnishing valuable insights to inform epidemic prevention and control efforts.

COVID-19 spreads across tourist system via tourists, causing a tremendous shock to the global tourism industry [29]. Due to the traffic limitation both home and abroad [3], tourism in all places has fallen into a torpor, greatly influencing the local tourism services and business activities [30]. Under the impact of COVID-19, in 2020 the number of international tourists dropped by 74%, export proceeds decreased by 1.3 trillion dollars, and 100 million to 120 million tourism jobs were confronted with a risk [5]. It has become highly challenging for cities with well-developed tourism to choose between epidemic control and economic development [31–33].

The virus propagation models established in the above-mentioned studies can well fit the epidemic transmission. Having considered the influences of the exposed and the characteristics of natality and mortality of population, the existing studies have used machine learning to implement virus propagation prediction. However, the presence of the asymptomatic and the incidence of population migration play an essential part in an actual transmission process. The current existing studies have explained the effectiveness of traffic control measures in blocking epidemic transmission and analyzed the blocking effects of different concrete control measures. In view of the impact of COVID-19 on society and economy, as well as the effectiveness of epidemic control, it is challenging to choose between epidemic control and economic development in order to avoid any harm of excessive epidemic prevention. It is quite important to promote the two-way dynamic coordination of epidemic prevention and control and economic development.

Considering the existing issues, this study will put forward an improved model to simulate the propagation of COVID-19, thus deriving the tendencies of epidemic transmission under distinct values of control intensity and reflecting the effectiveness of control measures in blocking epidemic transmission. To promote the two-way dynamic coordination of epidemic prevention and control and economic development, this study will employ the multi-objective optimization to balance the targets of epidemic prevention and control and of economic development, get an optimized value of traffic control intensity, and suggest scientific and reasonable traffic control measures.

## 3. Methodology

### 3.1 The study area and traffic control policies

This paper takes Xi’an City, China as the research object, considering that it possesses a booming tourism industry, while the generation of tourist behaviors would involve population migration, with which the highly infectious viruses would spread and cause a huge shock to the social economy of Xi’an. Due to the outbreak of COVID-19 in Xi’an, the government quickly took stringent control measures, even including lockdown control. The stringent control measures have effectively blocked epidemic transmission but impacted social production and livelihood. Therefore, taking Xi’an as a representative, this study explores the blocking effects of traffic control measures on epidemic transmission under distinct values of intensity, as well as feasible traffic control measures balancing the requirements for epidemic prevention and control and for economic development.

Xi’an, the capital of Shaanxi Province China, is a national central city. As of the end of 2021, the whole city of Xi’an boasted a permanent resident population of up to 13.163 million, with its regional GDP reaching 1.068828 trillion CNY and its output value of the tourism industry taking up 31% of the total output value [34], hence qualified as a tourist city. Xi’an was rated by China National Tourism Administration as a top tourist city of China [35], with tourism being its pillar industry with core competitiveness [36].

The new wave of COVID-19 in Xi’an started on December 9, 2021. The total lockdown control measure was implemented, starting from December 23, 2021. During the lockdown period, all long-distance passenger transport routes within the city territory went out of service, except that emergency vehicles were allowed to pass. Closed-off management was implemented in all communities (villages) and units within the whole city. The traffic management department tightened inspection at the transportation junctions, national and provincial highways within the city territory, external expressway intersections and other key sections, persuading personnel and vehicles to return that were taking an unnecessary departure from the downtown. It was not until January 24, 2022 that Xi’an lifted in the emergent state of total lockdown [37].

### 3.2 Data description and processing

#### 3.2.1 Virus propagation model data

The epidemic data in this paper were collected from December 9, 2021 to January 24, 2022 [38]. The data categories include the number of new cases, and the cumulative number of confirmed cases.

The permanent resident population in Xi’an was 13.163 million [34]. This study uses migration index data [39], which does not distinguish between modes of transportation and can reflect the size of the immigrant or emigrant population. This type of data were collected from December 9, 2021 to January 24, 2022. This study figures out the exact migrant population corresponding to the Baidu Migration index by formula [40] and uses this data for model building.

#### 3.2.2 Tourist economy data

According to the economic data from Xi’an Municipal Bureau of Statistics [34], COVID-19 has had minor impacts on primary and secondary industries but a substantial impact on tertiary industries, decreasing the growth rate to some degree. The tourism industry is the one that has been heavily shocked. As far as Xi’an City is concerned, the tourism industry comprised approximately 50% of its tertiary industries and represented roughly 31% of the total output value. Starting from 2020, under the impact of COVID-19, the output value of tourism has dropped by nearly 40%, while the output value of tertiary industries has increased by 6% on the whole. This indicates COVID-19 has had a significant impact on the tourism industry with great economic volume, though minor impacts on other industries than tourism among tertiary industries. While considering whatever traffic control measures to take during the epidemic period, governments shall give sufficient consideration to the core industrial status of tourism.

This study collects data from January 2011 to January 2019 from the tourism industry of Xi’an [34] and selects the data of the number of tourists received from home and abroad. A small amount of missing data is supplemented by nonlinear fitting.

The ARIMA model can predict the future tendency using historical time series data and can also be used to analyze and predict the tendency of time series, so this study adopts the ARIMA model to predict the tourism data. Given that the tourism data over this period were collected prior to the epidemic, the predicted results based on these data represent the tourism development status without control measures by January 2022. Due to the absence of tourist activities during the lockdown period, the function relationship between control intensity and the number of tourists received from home and abroad by January 2022 is derived according to the predicted results of tourism without control measures by January 2022 and the tourism data under the actual lockdown measure in the same month and year.

### 3.3 COVID-19 transmission dynamics modeling

#### 3.3.1 SEIQR model building with consideration of population migration value

Based on traditional SEIR virus propagation models, this study includes population migration, which occurs alongside epidemic transmission, in model building and extends the SEIR model by setting the exposed and the symptomatic infectious populations. The SEIQR model includes the susceptible (S), the exposed (E), the symptomatic infectious (I), the quarantined (Q) and the recovered (R) populations.

This model rests on the following hypotheses:

1. The susceptible (S) are a population vulnerable to virus, set as the permanent resident population.
2. The exposed (E) are a population of virus carriers with infective capacity within the infectious period and without apparent symptoms, including the asymptomatic.
3. The symptomatic infectious (I) are a population of virus carriers with infective capacity and related symptoms but unconfirmed.
4. The quarantined (Q) are a population without infective capacity who are treated in isolation after being confirmed.
5. The recovered (R) are a population who have been cured after being infected.
6. N(t) is the permanent resident population size of the city, leaving out the natality and mortality of the population while considering the daily immigration and emigration prior to traffic control measures.
7. N is the change of permanent resident population size of the city after a period of immigration and emigration, leaving out the natality and mortality of population, as well as the immigration and emigration prior to traffic control measures, in the model.

Considering that the exposed are infective, they may become the symptomatic infectious, or be immediately confirmed and quarantined with certain probability after undergoing virus detection. Some of the symptomatic infectious might mistake themselves for having a cold or other symptoms before being confirmed, thus having the probability to infect some other susceptible population. This paper assumes the confirmed have been quarantined without infective capacity. Table 1 shows an explanation of all parameters in the two phases of epidemic transmission.

**Table 1:** Explanation of parameters.

Parameter	Connotation	Parameter	Connotation
*S(t)*	size of population *S* on the *t*^th^ day	*β*_*i*,*1*_	infection rate of S in everyday contact with E under intensity *i*
*E(t)*	size of population *E* on the *t*^th^ day	*β*_*i*,*2*_	infection rate of S in everyday contact with I under intensity *i*
*I(t)*	size of population *I* on the *t*^th^ day	*α*_*1*_	probability of E becoming I
*Q(t)*	size of population *Q* on the *t*^th^ day	*α*_*2*_	probability of E being confirmed
*R(t)*	size of population *R* on the *t*^th^ day	*q*	probability of I being confirmed
*N(t)*	size of permanent resident population in the city on the *t*^th^ day	*δ*	recovery rate of Q
*IN*(*t*)	size of population immigrating into the city on the *t*^th^ day	*OUT*(*t*)	size of population emigrating from the city on the *t*^th^ day
*N*	size of permanent resident population in the city		

**Fig 1:**
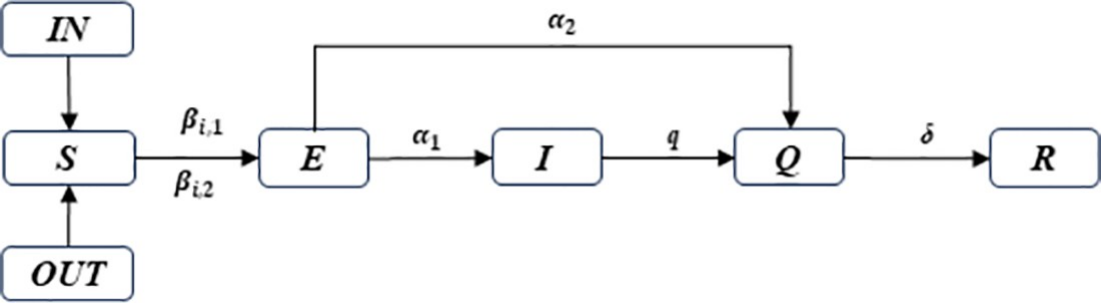
Diagram of SEIQR model without traffic control.

To depict the epidemic transmission without traffic control measures in the study area, population migration is also taken into consideration in model building, as shown in Fig 1. The iterative differential equations are set up as follows.


$$\frac{dS(t)}{dt}=-\frac{\beta_{\text{i},1}E(t)S(t)}{N}-\frac{\beta_{\text{i},2}I(t)S(t)}{N}+IN(t)-OUT(t)$$ (1)

$$\frac{dE(t)}{dt}=\frac{\beta_{\text{i},1}E(t)S(t)}{N}+\frac{\beta_{\text{i},2}I(t)S(t)}{N}-\alpha_{1}E(t)-\alpha_{2}E(t)$$ (2)

$$\frac{dI(t)}{dt}=\alpha_{1}E(t)-qI(t)$$ (3)

$$\frac{dQ(t)}{dt}=\alpha_{2}E(t)+qI(t)-\delta Q(t)$$ (4)

$$\frac{dR(t)}{dt}=\delta Q(t)$$ (5)

N(t)=S(t)+E(t)+I(t)+Q(t)+R(t) (6)

**Fig 2:**
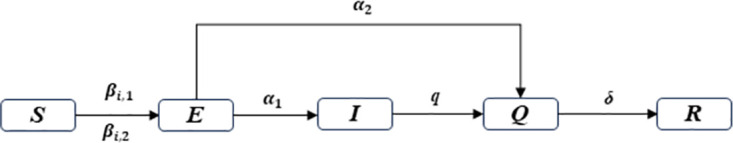
Diagram of SEIQR model under traffic control.

After adopting lockdown and other control measures in the study area, ignoring the incidence of population migration, other settings are consistent with the model settings prior to lockdown control, except for the change in permanent resident population in the city and other initial values of the model, as shown in Fig 2. The iterative differential equations are set up as follows.


$$\frac{dS(t)}{dt}=-\frac{\beta_{\text{i},1}E(t)S(t)}{N}-\frac{\beta_{\text{i},2}I(t)S(t)}{N}$$ (7)

$$\frac{dE(t)}{dt}=\frac{\beta_{\text{i},1}E(t)S(t)}{N}+\frac{\beta_{\text{i},2}I(t)S(t)}{N}-\alpha_{1}E(t)-\alpha_{2}E(t)$$ (8)

$$\frac{dI(t)}{dt}=\alpha_{1}E(t)-qI(t)$$ (9)

$$\frac{dQ(t)}{dt}=\alpha_{2}E(t)+qI(t)-\delta Q(t)$$ (10)

$$\frac{dR(t)}{dt}=\delta Q(t)$$ (11)

N=S(t)+E(t)+I(t)+Q(t)+R(t) (12)

#### 3.3.2 Model initialization and parameter estimation

In the selection of the initialization parameters of the model. The simulation of the virus propagation model in this paper falls into two phases. Phase I is one without control measures, and Phase II is one with control measures.

With respect to parameter selection for Phase I, starting with the first confirmed patient, we set the initial number of the infected and quarantined to be 1, assuming the probability of E becoming I is 0.1 [41], the probabilities of E and I being confirmed are 0.33 [42] and 0.5 [43], respectively, and the recovery rate of Q is 0.067 [44].

The initial values of all populations in Phase II are obtained after infection rate fitting and through simulation for Phase I. The size of population Q is 338, the size population R is 51, the size of population E is 410, and the size of population I is 46, whereby the initial size of population S in Phase II is derived. In Phase II, it is supposed that the probability of E turning into I is 0.2 [41], the probabilities of E and I being confirmed are 0.3 and 0.5, respectively, and the recovery rate of Q is 0.067, as shown in Table 2.

**Table 2:** Values of model parameters.

Parameter	Phase I	Phase II	Source
*β*_*i*,*1*_	0.684251124	0.384383447	Algorithmic fitting
*β*_*i*,*2*_	2.693425899	0.010029171	Algorithmic fitting
*α*_*1*_	0.1	0.2	[41]
*α*_*2*_	0.33	0.3	[42]
*q*	0.5	0.5	[43]
*δ*	0.067	0.067	[44]

In terms of estimation of infection rates. Considering that traffic control measures will generate an exponential scale effect on the change of population mobility intensity, this paper assumes that control intensity could have an exponential effect on epidemic transmission [45].

$$\beta_{i,s}=\beta_{0,s}\times e^{a\times \theta +b}$$ (13)

where the variable *θ* is the factor *traffic control intensity*, assumed to range within [0, 1]; *s* represents populations E and I with infective capacity, taking the value of 1 and 2, respectively; *β*_*0*,*s*_ denotes the infection rate of population *s* without control measures, and *β*_*i*,*s*_ denotes the infection rate of population *s* under traffic control intensity *i*.

This paper uses the Optuna framework to adjust and optimize the parameters. Optuna seeks a globally optimal solution within a high-dimensional hyper-parameter space, thereby minimizing the loss function of the machine learning model. It can effectively search the hyper-parameter space and accelerate the process of hyper-parameter adjustment and optimization. This paper performs model evolution via the assumed parameters *β*_*0*,*1*_ and *β*_*0*,*2*_ under the precondition that the initial values of other variables are given. In the Optuna framework of automatic hyper-parameter adjustment and optimization, by assuming the objective function, number of iterations and other key elements, the cumulative number of cases simulated by the model in each iteration process is compared with the truthful cumulative number of cases, and the root-mean-square (RMS) error between the two sets of data is calculated. After continuous training, the infection rate with the smallest error is recorded automatically. Finally, the infection rate parameter *β*_*0*,*s*_ before lockdown and infection rate parameter *β*_*1*,*s*_ during lockdown are obtained.

In this paper, the traffic control intensity without control measures is set as 0, and the traffic control intensity with the most stringent measures, such as lockdown, is set as 1. The final correlation can be determined as follows by solving the system of equations and, through the variation of control intensity, the infection rates of the two populations under the corresponding control intensity can be figured out.


$$\beta_{i,s}=\beta_{0,s}\times e^{-\theta \times In\frac{\beta_{0,s}}{\beta_{1,s}}}$$ (14)

### 3.4 The multi-objective optimization model balancing epidemic prevention and control and economic development

During the normalized epidemic-preventing process, epidemic prevention and control shall be coordinated with economic and social development. In order to promote the two-way dynamic sustainable development of epidemic prevention and control as well as of the economy and society, this paper adopts the method of multi-objective optimization to provide a scientific basis for epidemic control.

**Fig 3:**
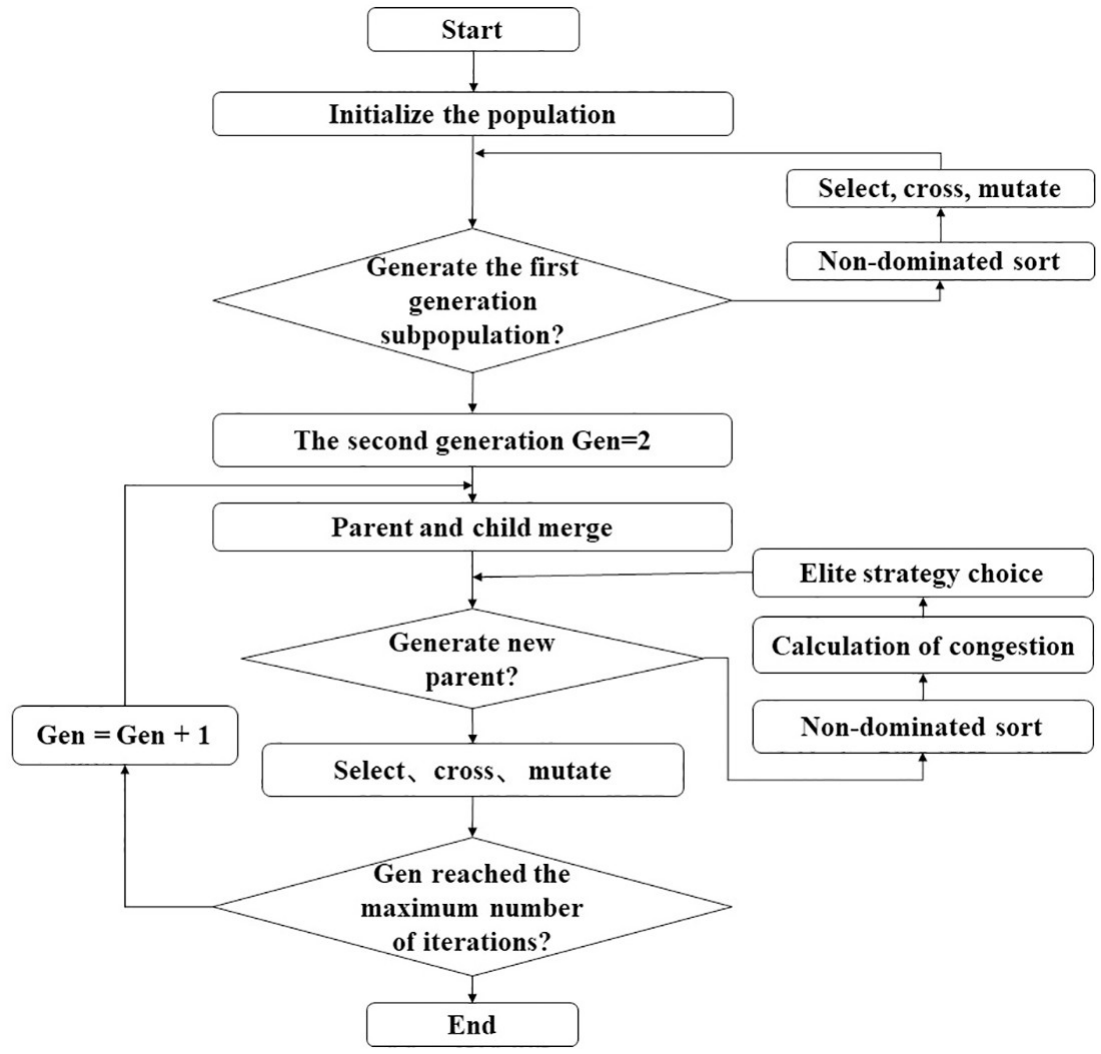
Algorithm flowchart.

This study adopts the multi-objective optimization algorithm NSGA-II. NSGA-II is a multi-objective genetic algorithm for solving multi-objective optimization problems, namely more than one single objective function needs to be considered in the optimization process. NSGA-II uses non-dominated sorting to sort solutions while allowing for the distribution of solutions to maintain the diversity of Pareto optimal sets. Since invented, this algorithm has received an extensive attention from scholars for its higher rate of convergence, stronger robustness, and more closeness to the real Pareto optimal front [46]. The concrete flow of the NSGA-II algorithm is shown in Fig 3.

This study determines two objective functions: traffic control intensity versus epidemic transmission and tourist quantity, respectively. In order to minimize the effect of epidemic transmission and promote the development of tourism during the control process, this paper uses the Pymoo library in Python for multi-objective optimization implementation and, by defining the objective functions and constraint conditions of the problem, selects the built-in NSGA-II optimization algorithm code to solve the problem, with other parameters and methods set in accordance to the built-in results of the Pymoo library.

The Pareto front and optimal solution set of the corresponding objective function are derived by the optimization algorithm NSGA-II. The Pareto optimal solution means there is no such a solution among all solutions that better satisfies all objectives simultaneously than the Pareto optimal solution. In the latter combinatorial optimization, the results corresponding to the two objectives are standardized before each of the objectives is allocated with a weight to calculate the result after multi-objective optimization.

## 4. Results

### 4.1 Estimation of infection rates

Given that other initial parameters of the model have been determined, the estimation of infection rates enables subsequent simulation of the virus propagation model.

In Phase I of epidemic development, considering the incidence of population migration and the infectiousness of population E, the basic parameters including the sizes of the five populations and recovery rate are determined as the initial values of the model for evolution. The infection rate of Phase I is derived by implementing the minimization of the RMS error between the presumed predicted data of model evolution and the actual data.

**Fig 4:**
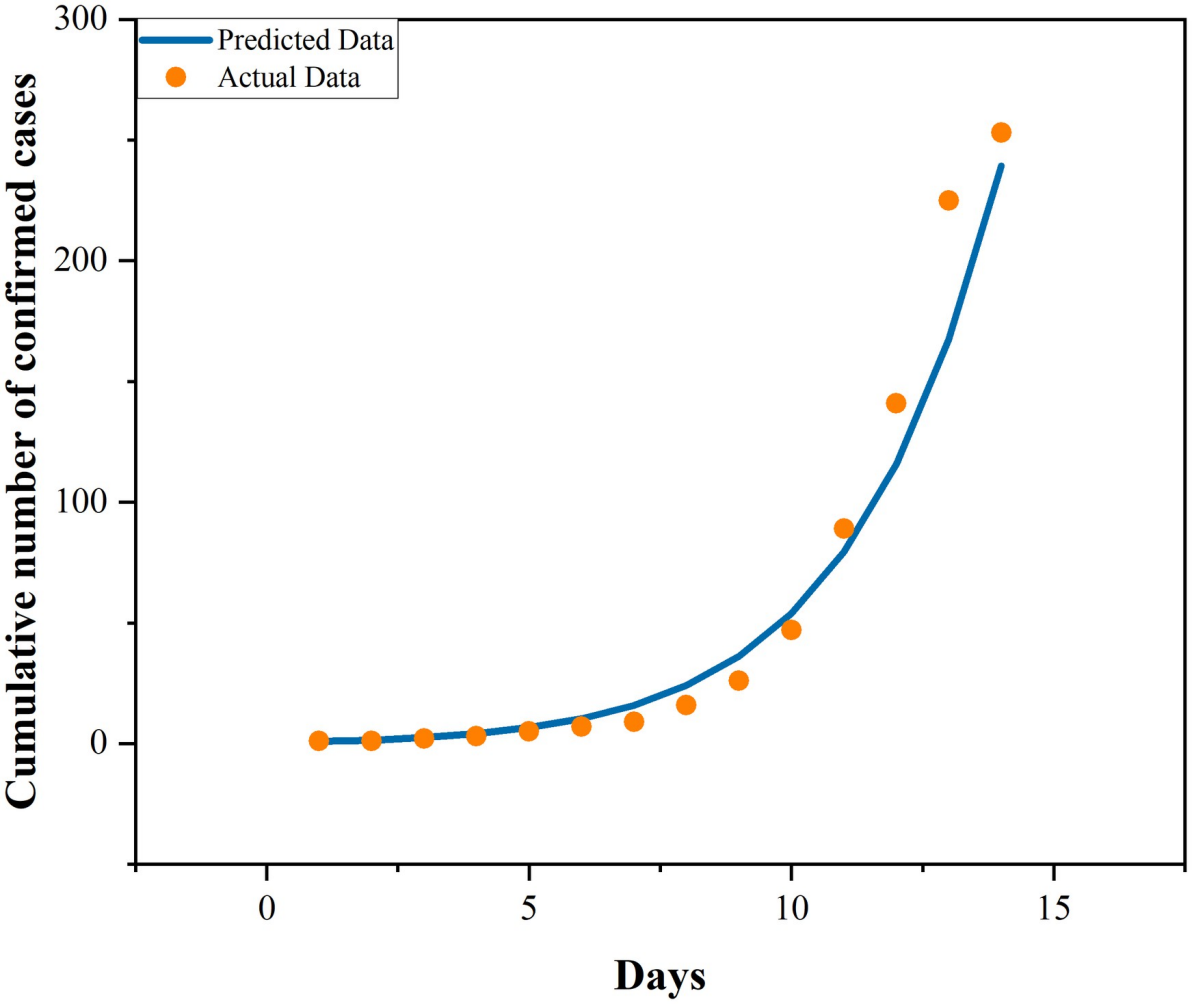
Fitting of cumulative number of cases in a pre-lockdown period.

**Fig 5:**
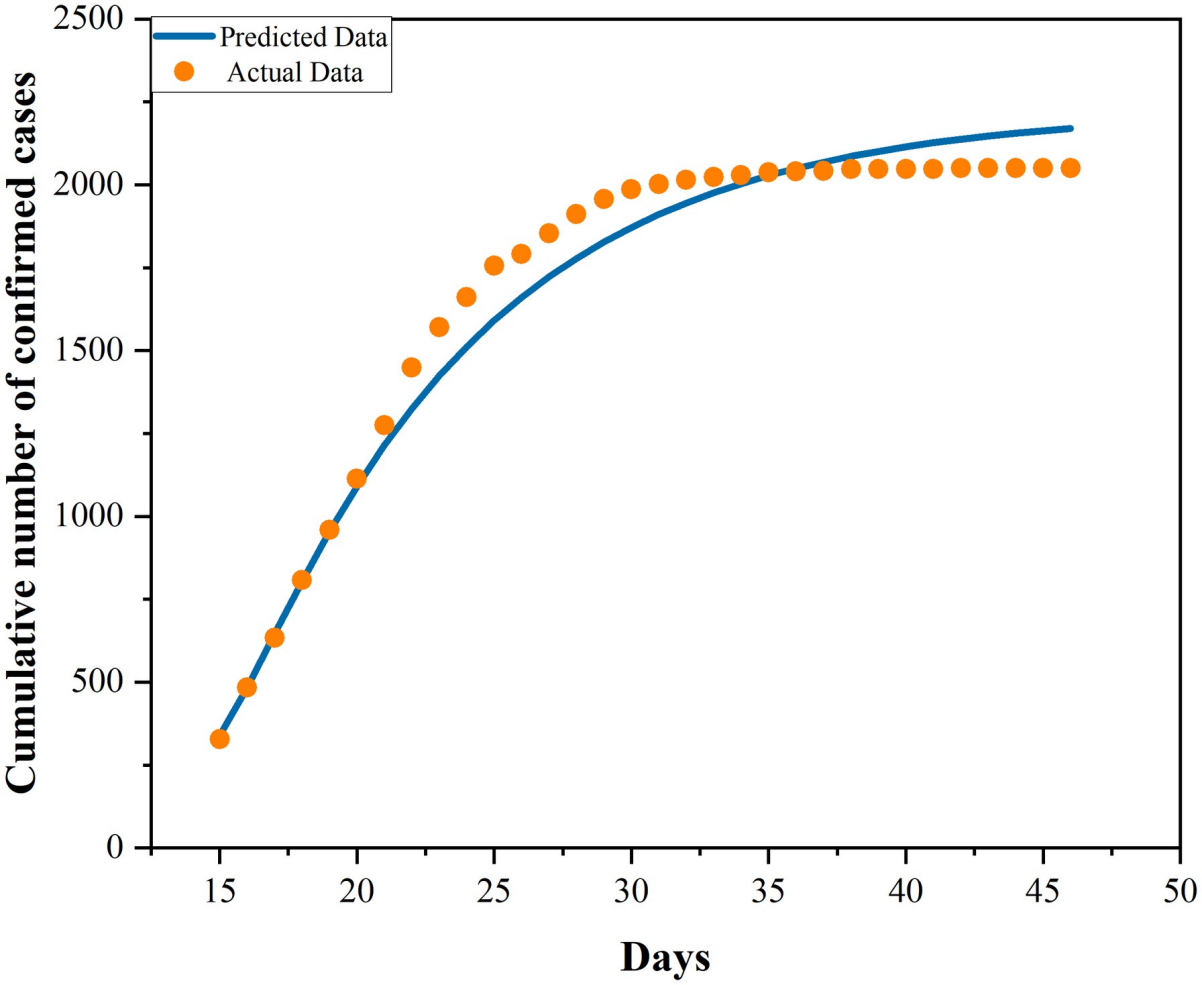
Fitting of cumulative number of cases in a post-lockdown period.

Since the government has adopted the control measure of lockdown in Phase II, leaving out population migration in this phase from the model and taking the results of model evolution in Phase I as the initial values of Phase II, the infection rate of Phase II is derived through minimization of the RMS error between the predicted data of the model and the actual data. It can be concluded that, owing to the adoption of control measures, the infection rate has declined dramatically, from 0.684251124 to 0.384383447 among population E, and from 2.693425899 to 0.010029171 among population I. This suggests traffic control measures facilitate blocking epidemic transmission. The exact parameter estimation effects of the two phases are presented in Figs 4 and 5. It is observable that the fitting conditions of the two phases are satisfactory.

### 4.2 Model validation

In order to validate the effectiveness of the model, this paper employs statistical analysis through correlation and regression analyses. We simulate using two preset virus propagation models before and after the lockdown measure to obtain the cumulative number of cases as the predicted data of them. Finally, a comparative analysis is conducted between the model-predicted data and actual cumulative number of cases.

**Fig 6:**
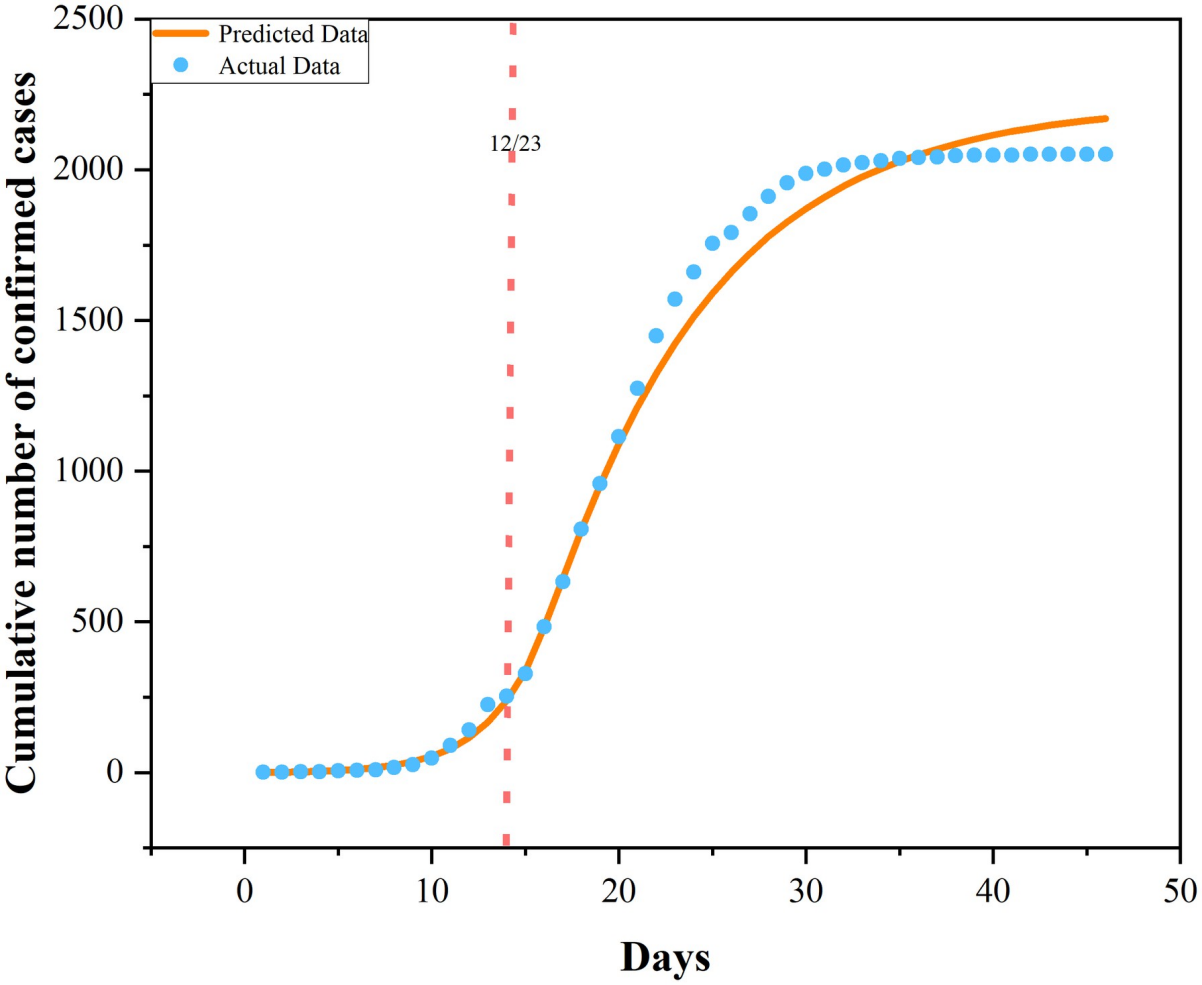
Fitting effect plot.

Table 3 illustrates that the Person correlation coefficient between the model-predicted cumulative number of cases and the actual cumulative number of cases is 0.996, the R-square in the regression analysis is 0.993, and the significance level is less than 0.001. The fitting effect diagram is shown in Fig 6. This means the fitting effect of the preset virus propagation models for the two phases is amazing, verifying the reliability of the preset models.

**Table 3:** Person correlation test.

	Person correlation	Significance level
Actual data	0.996	<0.001
Model predicted data	0.996	<0.001

### 4.3 Epidemic transmission under distinct values of control intensity

Following model validation, this paper sets six scenarios of epidemic transmission, each of which corresponds to a traffic control intensity of 0, 0.2, 0.4, 0.6, 0.8, and 1. Meanwhile, five traffic control levels are set to define the traffic control measures corresponding to distinct intervals of traffic control intensity [47]. See Table 4 for details.

**Table 4:** Description of traffic control measures.

Control level	Control intensity	Traffic control measures
1	(0.8, 1.0)	Lockdown management shall be practiced in medium- and high-risk areas of the city, including the implementation of closed-off management within residential compounds, suspension of urban public transportation services, and the implementation of traffic control measures on city roads, such as road closures, traffic flow restrictions, and the establishment of inspection checkpoints.
2	(0.6, 0.8]	Strict management shall be practiced in medium- and high-risk areas of the city, the community population mobility control measure shall be implemented, and public transport in medium- and high-risk areas shall go out of service.
3	(0.4, 0.6]	Temporary control measures shall be practiced on vital traffic thoroughfares or in crowded places, such as temporary traffic restrictions, lane closures, etc., and vehicles and personnel shall be inspected.
4	(0.2, 0.4]	Public transport ridership limitation shall be implemented within the city, vehicles shall be disinfected in time, and anti-epidemic inspection shall be conducted among all travelers at stations.
5	(0, 0.2]	Anti-epidemic inspection shall be conducted among travelers at traffic stations within the city.

Assuming the virus has not mutated and the medical technological level has not changed, let the most stringent lockdown measure be taken in Phase II. Then, the epidemic development in Xi’an would look as shown in the following figures. Overall, the peak value of the number of new cases per day would be controlled at 161, whereas the peak value of cumulative number of confirmed cases would reach 2224.

**Fig 7:**
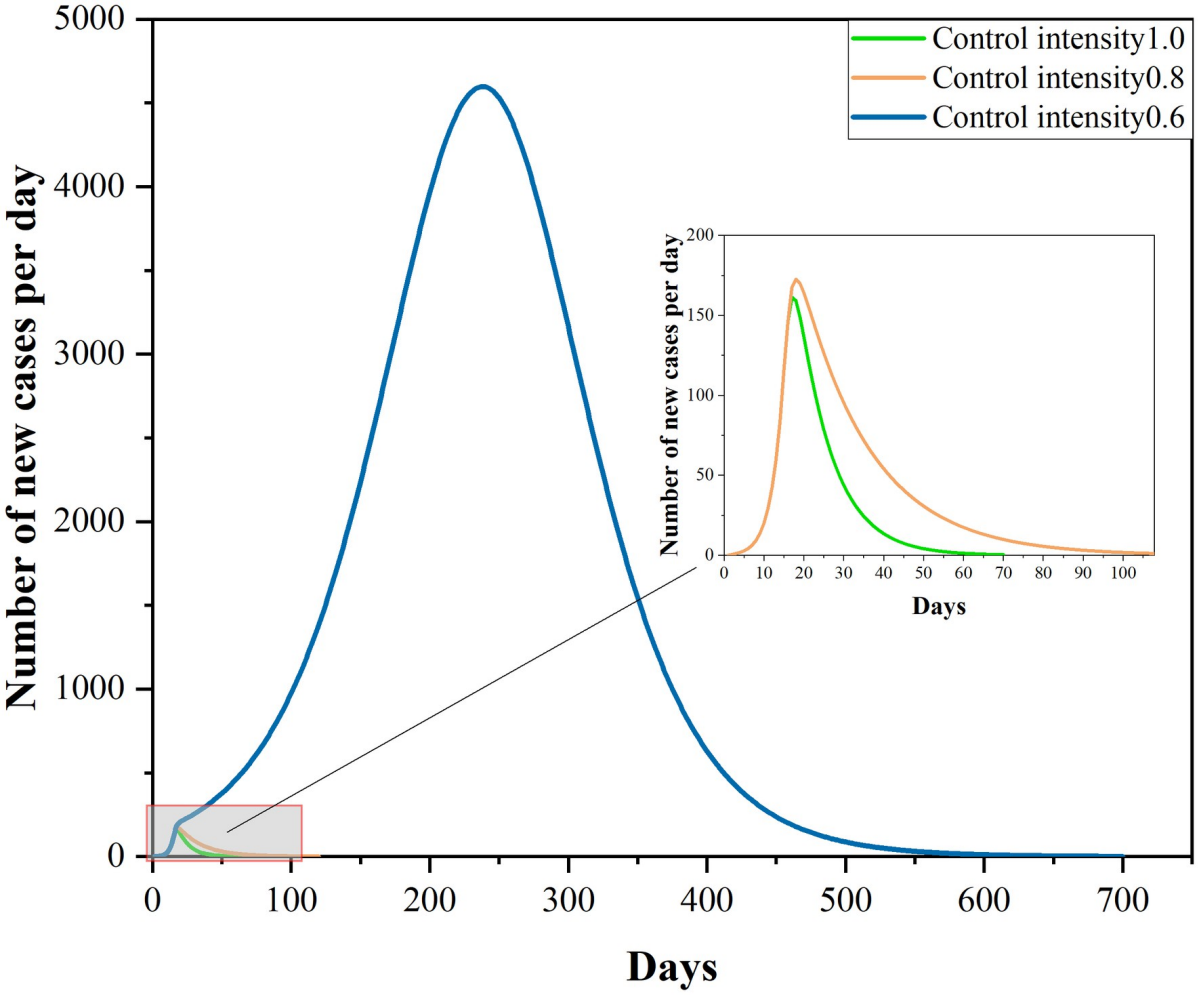
Plot of the number of new cases per day.

**Fig 8:**
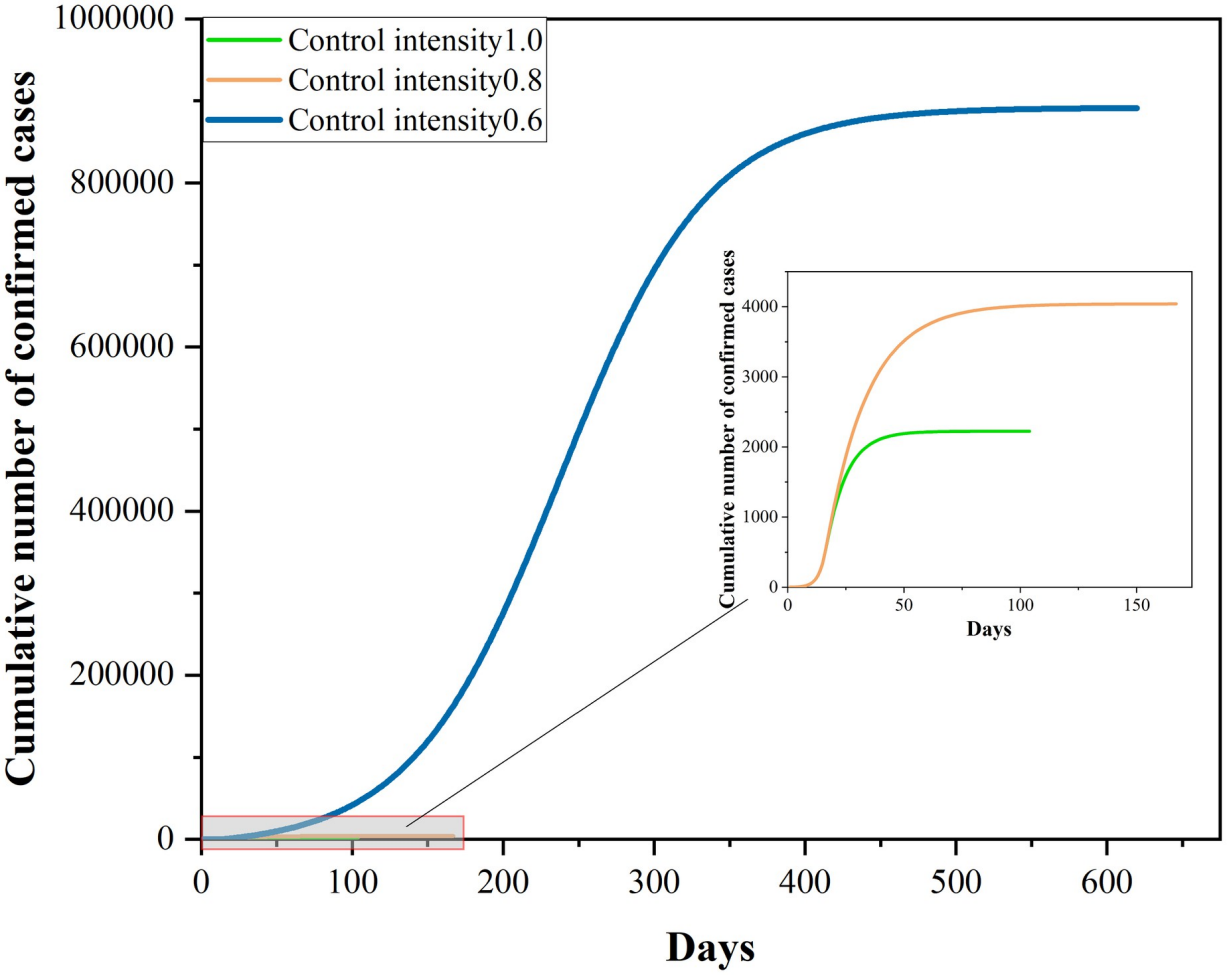
Plot of the cumulative number of confirmed cases.

The adoption of the lockdown control measure could cause a tremendous damage to the production, livelihood and economy of the city. Assuming the intensity of control measures is variable, this paper analyzes the epidemic transmission in Xi’an under distinct values of control intensity. Figs 7 and 8 reflect the epidemic development in the scenarios.

At the traffic control intensity of 0.8, the peak value of the number of new cases per day has reached 173, whereas the peak value of the cumulative number of confirmed cases has reached 4041, throughout the whole process of epidemic development. Compared to lockdown control, the peak values of the two targets have increased by 7.5% and 81.7%, respectively. At the control intensity of 0.6, the peak value of the number of new cases per day has risen to 4598, and the cumulative number of confirmed cases would rise to 891672. Under this control intensity the peak value of the number of new cases per day is 29 times that in the real situation, whereas the peak value of the cumulative number of confirmed cases is 401 times that in the real situation.

Assuming the traffic control intensity is 0 in Phase II, the peak value of the number of new confirmed cases per day would reach 1352601, and the peak value of the cumulative number of confirmed cases would reach 10562416. Compared to the lockdown control measure, the peak value of the number of new cases per day without control measures is 8401 times that in the real situation, whereas the peak value of the cumulative number of cases without control measures is 4749 times that in the real situation.

**Fig 9:**
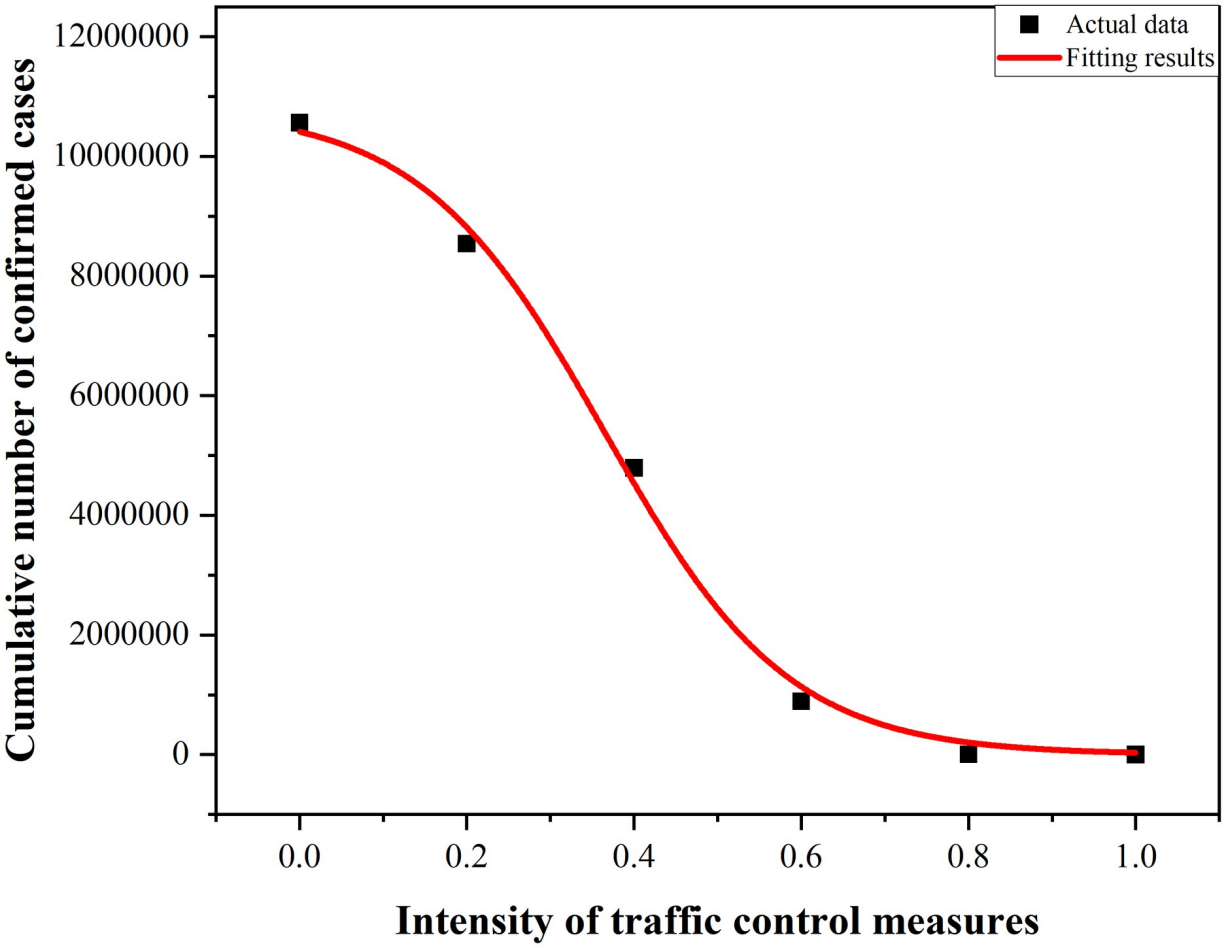
Function fitting plot.

After figuring out the data of cumulative number of confirmed cases in the six scenarios through the model, the cumulative number of confirmed cases under distinct control intensity levels undergoes nonlinear curve-fitting, and the function relationship between control intensity and cumulative number of confirmed cases is derived. The good fitting effects are observable in Fig 9. The specific fitting results are shown in Table 5.


y=10790700/(1+exp(9.08584×(x-0.36465))) (15)

**Table 5:** The fitting results.

Fitness statistics	Steady R^2^	R^2^	Chi-sqr tolerance
	0.99582	0.99749	up to 1E^-9^ level

### 4.4 Predicted results with respect to the tourist economy

**Fig 10:**
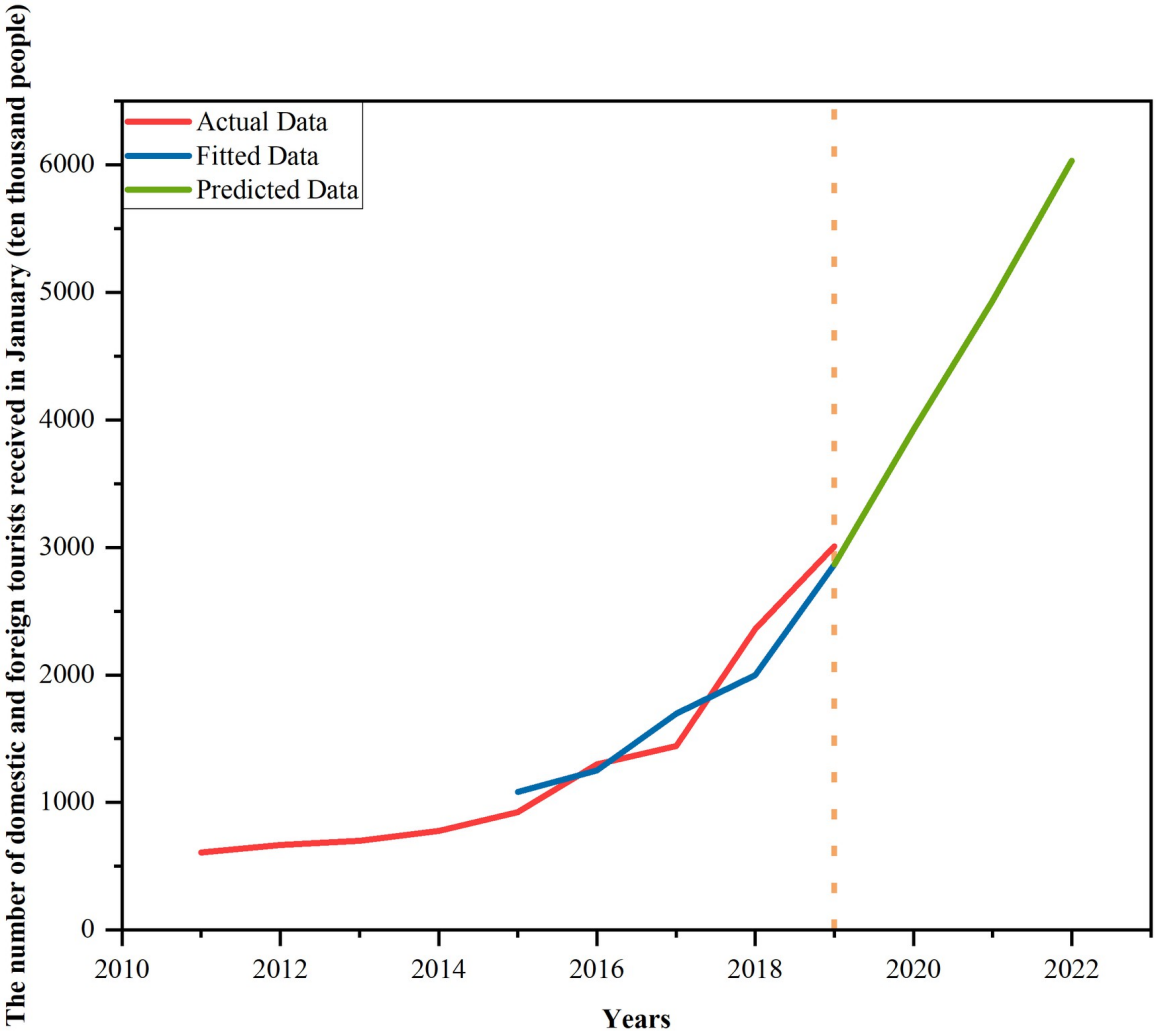
Plot of the predicted results.

In the previous data description section, the reason has been explained why the tourist economy is selected as a target. As an economic factor that needs considering in epidemic prevention and control, the selected data is the number of tourists received from home and abroad from 2011 to January 2019. By building the ARIMA model for model test, the parameters are finally selected as p = 2, d = 2, and q = 0. As shown in Table 6, the predicted results of the model meet the requirement. The number of tourists in Xi’an received from home and abroad is predicted to reach 60.3268 million by January 2022. The exact predicted results are shown in Fig 10.

**Table 6:** Predicted effect test.

Sample size	P value in ADF test	R^2^
9	<0.001	0.918

Based on the data from 2011 to 2019, this paper has predicted and derived the number of tourists received from home and abroad by January 2022 using the ARIMA model. As a critical index reflecting the tourist economy of Xi’an, this data represents the development trend of the tourist economy in Xi’an without the impact of epidemic and epidemic control measures. Meanwhile, due to the lockdown control measure taken in Xi’an, the actual number of tourists received from home and abroad by January 2022 was almost 0.

Considering that traffic control measures will generate an exponential scale effect on the change of population mobility intensity, this study assumes that control intensity could have an exponential effect on travel activities of tourists [45]. Consequently, this study, building upon existing data, establishes an exponential model between control intensity and the number of domestic and foreign tourists.


$$y=-6032.68\;\times \;e^{x\times {\text{log}}_{e}2}+12065.36$$ (16)

### 4.5 The multi-objective optimization results balancing epidemic prevention and control and economic development

**Fig 11:**
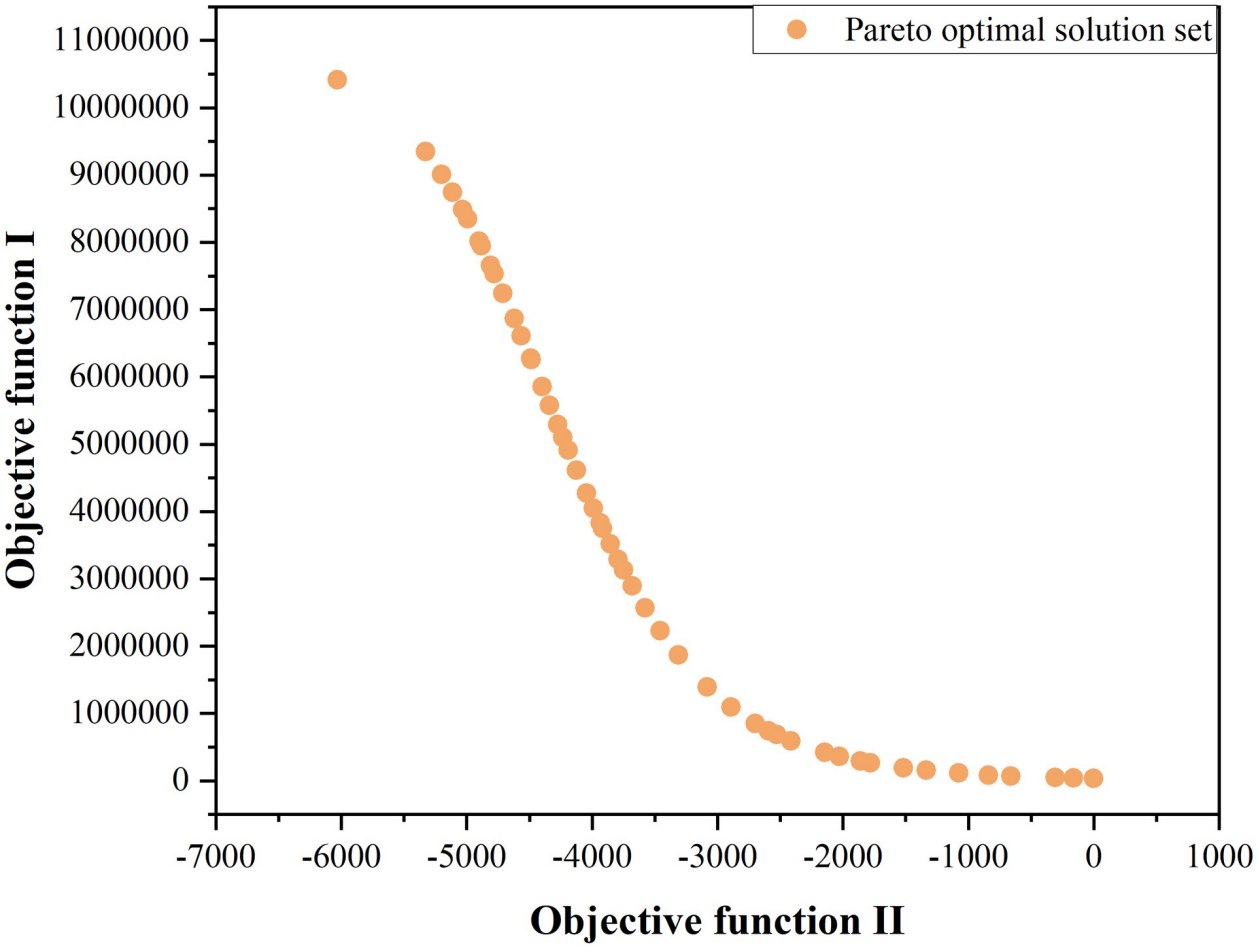
Pareto front plot.

This study has determined the function relationships between control intensity and cumulative number of cases, and between control intensity and number of tourists received from home and abroad by January 2022, and defined the objective functions to be optimized. By setting the one variable and the two objective functions, the objects of the problem, the concrete parameters and other conditions are created, and multi-objective optimization is implemented by the NSGA-II algorithm using Python. In model building, the constraint range of the variable is assumed to be [0, 1], the population size and the progeny size are 50 each, and the number of iterations is 200, with the other parameters and methods set in accordance to the built-in results of the Pymoo library. The corresponding Pareto front and Pareto optimal solution set after balanced optimization are obtained through calculation by the NSGA-II algorithm. The results are shown in Fig 11.

**Fig 12:**
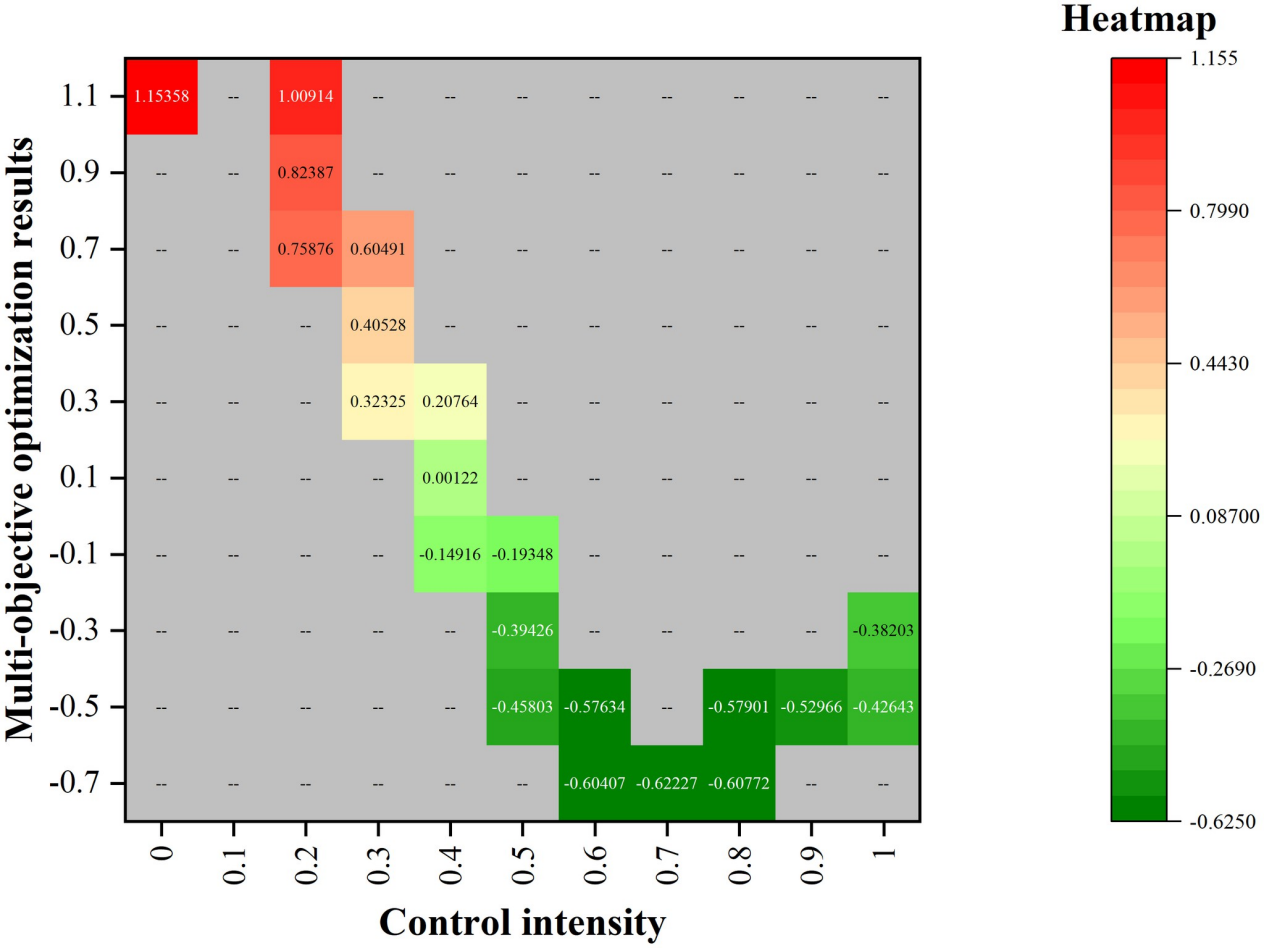
Heat map of multi-objective optimization results.

Which particular solution to select from among the Pareto optimal solutions depends on the needs of practical application or decision-maker’s goal. In this paper, the approach is to assign a weight to each objective to select the optimal solution. Concerning the solutions in the Pareto solution set obtained by the NSGA-II algorithm, the first step is to standardize the values of the two objective functions corresponding to the solutions in the Pareto solution set to eliminate the dimensional difference between the values. Next, considering the relative importance of epidemic prevention and control and economic development, the weights of objective function 1 and objective function 2 are set to 0.764 and 0.236 respectively according to the entropy weight method. Since the smaller the expected results the better, the optimal solution of traffic control intensity through calculation is 0.68, as shown in Fig 12. According to the choice of traffic control intensity, a series of control measures such as strict management in Medium- and high-risk areas of the city, implementation of population flow control measures in communities, and suspension of public transportation in Medium- and high-risk areas in the city can be adopted. Taking traffic control measures under this intensity is beneficial to balancing the requirements for epidemic prevention and control and for economic development.

## 5. Discussion

Given the effectiveness of traffic control measures in pandemic containment and the need to mitigate the socio-economic impacts of unreasonable control measures, it becomes crucial to foster a bidirectional dynamic synergy between pandemic control and economic development. This study begins by proposing an improved SEIQR model to explore the pandemic’s progression under varying levels of traffic control intensity. Our findings reveal a substantial decrease in the cumulative case count as traffic control intensity rises across different simulation scenarios. The escalation in traffic control intensity effectively curtails epidemic spread, but it necessitates the consideration of the adverse consequences associated with high-intensity traffic restrictions. Subsequently, to strike a balance between the demands of pandemic control and economic development, this research employs a multi-objective optimization approach. Through a combination optimization approach, we identify scientifically suitable traffic control intensities and provide specific recommendations for traffic control measures. This approach facilitates the harmonization of pandemic control and economic development objectives, offering valuable guidance for future government initiatives in pandemic control and emergency response efforts.

This study is distinguished from the previous ones. In the aspect of epidemic models, it has given further consideration to the presence of the asymptomatic and the incidence of population migration based on the SEIR model built by Gopal et al [48]. and the machine learning model proposed by Mohanraj et al [49]. Moreover, the model is accurate enough to simulate epidemic transmission, thus disclosing the blocking effects of traffic control measures on COVID-19 under distinct values of intensity and predicting the propagation of COVID-19 in Xi’an City under distinct levels of control intensity.

In the aspect of balancing epidemic prevention and control and economic development, in view of previous studies focusing on the effectiveness of traffic control measures in blocking the epidemic [27,50], the equally unreasonable epidemic prevention and control measures could negatively impact the society and economy [13]. Shi et al. only referred to the economic loss in epidemic control as a policy reference [51]. In order to avoid any harm due to excessive epidemic prevention, however, this paper has balanced the requirements for epidemic prevention and control and for social and economic development and offered scientific and reasonable control measures using the multi-objective optimization method. The research results of this paper can provide a reference for governments to formulate scientific and reasonable control strategies in the future, as well as a scientific basis for new development in epidemic prevention and control and emergency treatment.

In the aspect of universality of the research methodology, the research object of this paper is ‘tourist cities’, which refers collectively to a class of cities that possess natural landscapes or humanistic connotations, historical profundities and other unique resources, with a tourism output value exceeding the urban GDP by 7%. Such classes are prone to tourist behaviors, exhibiting distinct features of population migration. During specific seasons and holidays, their tourist economy tends to be more booming than usual. Taking London in the UK, the Balearic Islands in Spain, and other famous tourist destinations as examples, the cities typically have booming tourism and significant seasonal population mobility [52,53]. As the traffic control state of the research object and the considered economic factor have been determined, the idea and methodology of this study apply to other international tourist cities and can provide generalized policy suggestions for them.

Based on the research in this article, we will put forward some suggestions for epidemic prevention and control. In view of the high infectiousness of virus, related departments usually would take stringent control measures to curb epidemic transmission. This played a great part in the earlier stage of the epidemic. However, with the change of the epidemic prevention situation, overly stringent control measures could trigger a contradiction between society, economy and people’s production and livelihood. Excessive epidemic prevention is inadvisable. Related departments should give overall consideration to the social and economic impacts of control and suggest scientific policy measures to promote epidemic prevention and control as well as social and economic development. Meanwhile, based on scientific research, epidemic control measures should change flexibly with the change of the epidemic prevention situation instead of staying unchanged, which will do good to social functioning and people’s production and livelihood.

There exists some scope for improvement in this study. In the aspect of model building, due to the lack of data on vaccination, age, and other types, we have assumed the susceptible population is uniformly infectable. However, the effect of viruses, in reality, tends to differ by age [54]. In future model building, the susceptible population can be subdivided in such a way that the modeling would conform better to the real condition. Due to the limited availability and scarcity of publicly accessible data concerning the number of Chinese tourists, we were unable to obtain additional official government-provided data. This study has been improved based on relevant literature. The accuracy of our analysis would be enhanced if corresponding data becomes accessible in the future. Meanwhile, this study has proposed corresponding traffic control measures recommendations for the optimal traffic control intensity. In future research, algorithms or models can be designed based on specific traffic control measures to explore the effectiveness of various traffic control strategies.

## 6. Conclusion

Infectious diseases have existed in the history of human development and have always been one of the challenges to human survival and development. Effective and reasonable prevention and control of virus spread is a major issue related to social development and people’s production and life. This study introduces the SEIQR model, which takes into account population migration, the characteristics of latent carriers, and asymptomatic infections. It incorporates levels of control intensity into the construction of the SEIQR model to simulate the spread of the epidemic under varying degrees of traffic control. Once the objective relationships are established, NSGA-II algorithm is employed for multi-objective optimization of the two objective functions, resulting in pareto optimal solution set that balances the two objective functions. Through weighted combination optimization, an appropriate level of traffic control intensity is determined. The primary conclusions are as follows:

1. The improved SEIQR model exhibits a high degree of accuracy and a strong fitting performance in the case study, with a Person correlation coefficient of 0.996 and a R-square of 0.993.
2. A higher level of traffic control intensity in different simulation scenarios can significantly enhance the epidemic containment effect, but cause higher socio-economic losses for tourism cities. The reasonable traffic control intensity explored in this study is 0.68.
3. The government can adopt a series of control measures such as strict management in the medium and high-risk areas of the city, implementation of measures to control population movement within residential neighborhoods, and suspension of public transport in the medium and high-risk areas.

These traffic control measures strike a balance between pandemic management and economic development, mitigating the socio-economic repercussions of unreasonable control intensity. They facilitate the restoration and operation of social production and life, fostering a bidirectional synergy between pandemic control and economic development. The insights and findings of this study provide a scientific basis for future government strategies in pandemic control and contribute to the development of effective control measures.

## Supporting information

S1 Data (ZIP)

## References

[1] Xi Z, Min-Peng C. Adaptation and green recovery: synergistic responses to the COVID-19 pandemic and climate compounding risks. Advances in Climate Change Research. 2022;18(6):720.

[2] Kumar V, Alshazly H, Idris SA, Bourouis S. Evaluating the Impact of COVID-19 on Society, Environment, Economy, and Education. SUSTAINABILITY. 2021;13(24):13642.

[3] Varzaru AA, Bocean CG, Cazacu M. Rethinking Tourism Industry in Pandemic COVID-19 Period. SUSTAINABILITY. 2021;13(12).

[4] Tao F. DEA model based research on the tour efficiency in central region cities of China. M.Sc. Thesis, Nanchang University. 2017.

[5] Matsuura T, Saito H. The COVID-19 pandemic and domestic travel subsidies. ANNALS OF TOURISM RESEARCH. 2022;92. doi: 10.1016/j.annals.2021.103326 34815608 PMC8602970

[6] Duro JA, Perez-Laborda A, Turrion-Prats J, Fernandez-Fernandez M. Covid-19 and tourism vulnerability. TOURISM MANAGEMENT PERSPECTIVES. 2021;38. doi: 10.1016/j.tmp.2021.100819 34873568 PMC8635291

[7] Zhang Q, Yang H. Geovisualizing the changes in metro passenger flows of Kunming under the impact of COVID-19. Journal of Transport Geography. 2022;104:103420. doi: 10.1016/j.jtrangeo.2022.103420 35992219 PMC9382435

[8] Kim J, Kwan M-P. The impact of the COVID-19 pandemic on people’s mobility: A longitudinal study of the U.S. from March to September of 2020. Journal of Transport Geography. 2021;93:103039. doi: 10.1016/j.jtrangeo.2021.103039 36569218 PMC9759208

[9] Xiang W, Chen L, Wang B, Xue Q, Hao W, Liu X. Policies, population and impacts in metro ridership response to COVID-19 in Changsha. JOURNAL OF TRANSPORTATION SAFETY & SECURITY. 2022;14(11):1955–75.

[10] Zhao Ning LIU D. Social Prevention and Information Disclosure: Evolution Mechanism and Prediction Model of Severe Infectious Diseases. Operations Research and Management Science. 2023;32(1):121–6.

[11] Lou J, Shen X, Niemeier D. Are stay-at-home orders more difficult to follow for low-income groups? Journal of Transport Geography. 2020;89:102894. doi: 10.1016/j.jtrangeo.2020.102894 33519126 PMC7832451

[12] He W, Chen Q. Progress in source tracking of SARS-CoV-2. Nan Fang Yi Ke Da Xue Xue Bao. 2020;40(12):1838–42. doi: 10.12122/j.issn.1673-4254.2020.12.22 33380405 PMC7835685

[13] Yu C. An explorative analysis into China’s general policy of dynamic zero COVID-19 in epidemic prevention and control: abiding by Xi Jinping’s important discourse on coordinating the COVID-19 response with economic and social development. Theory Construction. 2022;38(04):26–33.

[14] Li F, Li YY, Liu MJ, Fang LQ, Dean NE, Wong GWK, et al. Household transmission of SARS-CoV-2 and risk factors for susceptibility and infectivity in Wuhan: a retrospective observational study. LANCET INFECTIOUS DISEASES. 2021;21(5):617–28. doi: 10.1016/S1473-3099(20)30981-6 33476567 PMC7833912

[15] Kermack WO, McKendrick AG. Contributions to the mathematical theory of epidemics—I. 1927. Bulletin of mathematical biology. 1991;53(1–2):33–55. doi: 10.1007/BF02464423 2059741

[16] Ding Y, Wandelt S, Sun X. TLQP: Early-stage transportation lock-down and quarantine problem. TRANSPORTATION RESEARCH PART C-EMERGING TECHNOLOGIES. 2021;129:103218. doi: 10.1016/j.trc.2021.103218 36313400 PMC9587919

[17] Kondo K. Simulating the impacts of interregional mobility restriction on the spatial spread of COVID-19 in Japan. SCIENTIFIC REPORTS. 2021;11(1):1–15. 34556681 10.1038/s41598-021-97170-1 PMC8460743

[18] Qian X, Ukkusuri SV. Connecting urban transportation systems with the spread of infectious diseases: A Trans-SEIR modeling approach. TRANSPORTATION RESEARCH PART B-METHODOLOGICAL. 2021;145:185–211.

[19] Li MY, Muldowney JS. Global stability for the SEIR model in epidemiology. Mathematical biosciences. 1995;125(2):155–64. doi: 10.1016/0025-5564(95)92756-5 7881192

[20] Smirnova A, deCamp L, Chowell G. Forecasting Epidemics Through Nonparametric Estimation of Time-Dependent Transmission Rates Using the SEIR Model. BULLETIN OF MATHEMATICAL BIOLOGY. 2019;81(11):4343–65. doi: 10.1007/s11538-017-0284-3 28466232

[21] Xiang W, Chen L, Peng QJ, Wang B, Liu XB. How Effective Is a Traffic Control Policy in Blocking the Spread of COVID-19? A Case Study of Changsha, China. INTERNATIONAL JOURNAL OF ENVIRONMENTAL RESEARCH AND PUBLIC HEALTH. 2022;19(13). doi: 10.3390/ijerph19137884 35805541 PMC9265603

[22] Wang HS, Ma JL, Zhao ZP, Jia ZY, Ji ZY, Wu J. Adversarial Training for Predicting the Trend of the COVID-19 Pandemic. JOURNAL OF DATABASE MANAGEMENT. 2022;33(1).

[23] Jia WP, Han K, Song Y, Cao WZ, Wang SS, Yang SS, et al. Extended SIR Prediction of the Epidemics Trend of COVID-19 in Italy and Compared With Hunan, China. FRONTIERS IN MEDICINE. 2020;7. 10.3389/fmed.2020.00169 PMC7218168 32435645

[24] Nishi A, Lee LF, Tsuji H, Takasaki Y, Young SD. Revisiting the county/city-level event risk assessment during the COVID-19 pandemic. JOURNAL OF INFECTION. 2021;82(5):191–2. doi: 10.1016/j.jinf.2020.12.031 33406395 PMC8455339

[25] Li D-J, Ko N-Y, Chang Y-P, Yen C-F, Chen Y-L. Mediating Effects of Risk Perception on Association between Social Support and Coping with COVID-19: An Online Survey. INTERNATIONAL JOURNAL OF ENVIRONMENTAL RESEARCH AND PUBLIC HEALTH. 2021;18(4). doi: 10.3390/ijerph18041550 33561974 PMC7915796

[26] Zhong Y, Liu W, Lee T-Y, Zhao H, Ji J. Risk perception, knowledge, information sources and emotional states among COVID-19 patients in China. NURSING OUTLOOK. 2021;69(1):13–21. 32980153 10.1016/j.outlook.2020.08.005 PMC7442898

[27] Xing-li JIA, Wu-xiao Z, Xing-jia HAN, Meng-hua YAN, Xue-fang QIN. Blocking effects of traffic control measures on COVID-19 transmission in city territories. China Journal of Highway and Transport. 2022;35(1):252.

[28] Xiang W, Chen L, Yan X, Wang B, Liu X. The impact of traffic control measures on the spread of COVID-19 within urban agglomerations based on a modified epidemic model. CITIES. 2023;135. doi: 10.1016/j.cities.2023.104238 36817574 PMC9922589

[29] Aldao C, Blasco D, Espallargas MP, Rubio SP. Modelling the crisis management and impacts of 21st century disruptive events in tourism: the case of the COVID-19 pandemic. TOURISM REVIEW. 2021;76(4):929–41.

[30] Klinsrisuk R, Pechdin W. Evidence from Thailand on Easing COVID-19’s International Travel Restrictions: An Impact on Economic Production, Household Income, and Sustainable Tourism Development. SUSTAINABILITY. 2022;14(6).

[31] Haroon O, Ali M, Khan A, Khattak MA, Rizvi SAR. Financial market risks during the COVID-19 pandemic. Emerging Markets Finance and Trade. 2021;57(8):2407–14.

[32] Liu K. COVID-19 and the Chinese economy: impacts, policy responses and implications. International Review of Applied Economics. 2021;35(2):308–30.

[33] Zhang D, Hu M, Ji Q. Financial markets under the global pandemic of COVID-19. FINANCE RESEARCH LETTERS. 2020;36. doi: 10.1016/j.frl.2020.101528 32837360 PMC7160643

[34] Xi’an Statistical Bureau. Statistical Data. http://tjj.xa.gov.cn/tjsj/tjsj/ndsj/2021/1.html.

[35] Shaanxi Provincial People’s Government. Provincial Situation. http://www.shaanxi.gov.cn/sq/dsjj/xas_4665/202008/t20200827_1302228.html.

[36] Xi’an Municipal People’s Government. Policy Documents. https://www.xa.gov.cn/ztzl/ztzl/lwlbzt/zcwj/619468c5f8fd1c0bdc69cced.html.

[37] Lingshan L. Traffic control effect analysis and control point selection strategy optimization in epidemic transmission. M.Sc. Thesis, Xihua University. 2022.

[38] Shaanxi Provincial Health Care Commission. Health and Wellness News. http://sxwjw.shaanxi.gov.cn/sy/wjyw/index_42.html.

[39] Baidu Migration. Baidu Migration Index. https://qianxi.baidu.com/#/.

[40] Cong Wang, Jie Y. An Inversion of the Constitution of the Baidu Migration Scale Index. Journal of University of Electronic Science and Technology of China. 2021;50(4):616–26.

[41] Lauer SA, Grantz KH, Bi QF, Jones FK, Zheng QL, Meredith HR, et al. The Incubation Period of Coronavirus Disease 2019 (COVID-19) From Publicly Reported Confirmed Cases: Estimation and Application. ANNALS OF INTERNAL MEDICINE. 2020;172(9):577-+. doi: 10.7326/M20-0504 32150748 PMC7081172

[42] Linton NM, Kobayashi T, Yang Y, Hayashi K, Akhmetzhanov AR, Jung S-m, et al. Incubation Period and Other Epidemiological Characteristics of 2019 Novel Coronavirus Infections with Right Truncation: A Statistical Analysis of Publicly Available Case Data. JOURNAL OF CLINICAL MEDICINE. 2020;9(2). 10.3390/jcm9020538 PMC7074197 32079150

[43] Ding Ying Z J, Yang Mu, Gong Peng, Jia Lipeng, Deng Shaocun. Communicable disease transmission model for the prevention and control of COVID-19 in Wuhan City, China. Journal of Tsinghua University (Science and Technology). 2021(12):1452–61.

[44] Zhou W, Bai D, Zhao J. Modeling and Analysis of COVID-19 in Wuhan. Complex Systems and Complexity Science. Complex Systems and Complexity Science. 2020;17(4):58–65.

[45] Liu S, Yamamoto T. Role of stay-at-home requests and travel restrictions in preventing the spread of COVID-19 in Japan. TRANSPORTATION RESEARCH PART A-POLICY AND PRACTICE. 2022;159:1–16. doi: 10.1016/j.tra.2022.03.009 35309690 PMC8920346

[46] Deb K, Pratap A, Agarwal S, Meyarivan T. A fast and elitist multiobjective genetic algorithm: NSGA-II. IEEE transactions on evolutionary computation. 2002;6(2):182–97.

[47] Zhang X-L, Zhang Z-H, Xu Z-J, Li G, Sun Q, Hou X-J. Sea ice disasters and their impacts since 2000 in Laizhou Bay of Bohai Sea, China. NATURAL HAZARDS. 2013;65(1):27–40.

[48] Gopal R, Chandrasekar VK, Lakshmanan M. Analysis of the second wave of COVID-19 in India based on SEIR model. EUROPEAN PHYSICAL JOURNAL-SPECIAL TOPICS. 2022;231(18–20):3453–60. doi: 10.1140/epjs/s11734-022-00426-8 35039761 PMC8756415

[49] Mohanraj G, Mohanraj V, Marimuthu M, Sathiyamoorthi V, Luhach AK, Kumar S. Epidemic Prediction using Machine Learning and Deep Learning Models on COVID-19 Data. JOURNAL OF EXPERIMENTAL & THEORETICAL ARTIFICIAL INTELLIGENCE. 2023;35(3):377–93.

[50] Borkowski P, Jażdżewska-Gutta M, Szmelter-Jarosz A. Lockdowned: Everyday mobility changes in response to COVID-19. Journal of Transport Geography. 2021;90:102906. doi: 10.1016/j.jtrangeo.2020.102906 35721765 PMC9188832

[51] Shi Y, Huang R, Cui H. Prediction and Analysis of Tourist Management Strategy Based on the SEIR Model during the COVID-19 Period. INTERNATIONAL JOURNAL OF ENVIRONMENTAL RESEARCH AND PUBLIC HEALTH. 2021;18(19). doi: 10.3390/ijerph181910548 34639848 PMC8508590

[52] Fernandez-Morales A, Cisneros-Martinez JD, McCabe S. Seasonal concentration of tourism demand: Decomposition analysis and marketing implications. TOURISM MANAGEMENT. 2016;56:172–90.

[53] Martin JMM, Martin JAR, Mejia KAZ, Fernandez JAS. Effects of Vacation Rental Websites on the Concentration of Tourists-Potential Environmental Impacts. An Application to the Balearic Islands in Spain. INTERNATIONAL JOURNAL OF ENVIRONMENTAL RESEARCH AND PUBLIC HEALTH. 2018;15(2). 10.3390/ijerph15020347 PMC5858416 29462863

[54] Sangiorgio V, Parisi F. A multicriteria approach for risk assessment of Covid-19 in urban district lockdown. SAFETY SCIENCE. 2020;130. doi: 10.1016/j.ssci.2020.104862 32536749 PMC7275161

